# Supplementary material for: Evidence supported by Mendelian randomization: impact on inflammatory factors in knee osteoarthritis
Source: Front Med (Lausanne). 2024 May 28;11:1382836. doi: 10.3389/fmed.2024.1382836 (PMC11165061; doi:10.3389/fmed.2024.1382836)
Supplement: Supplementary file 1 [file Data_Sheet_1.ZIP › data/GCST90274784_5e6/784.forest.pdf]

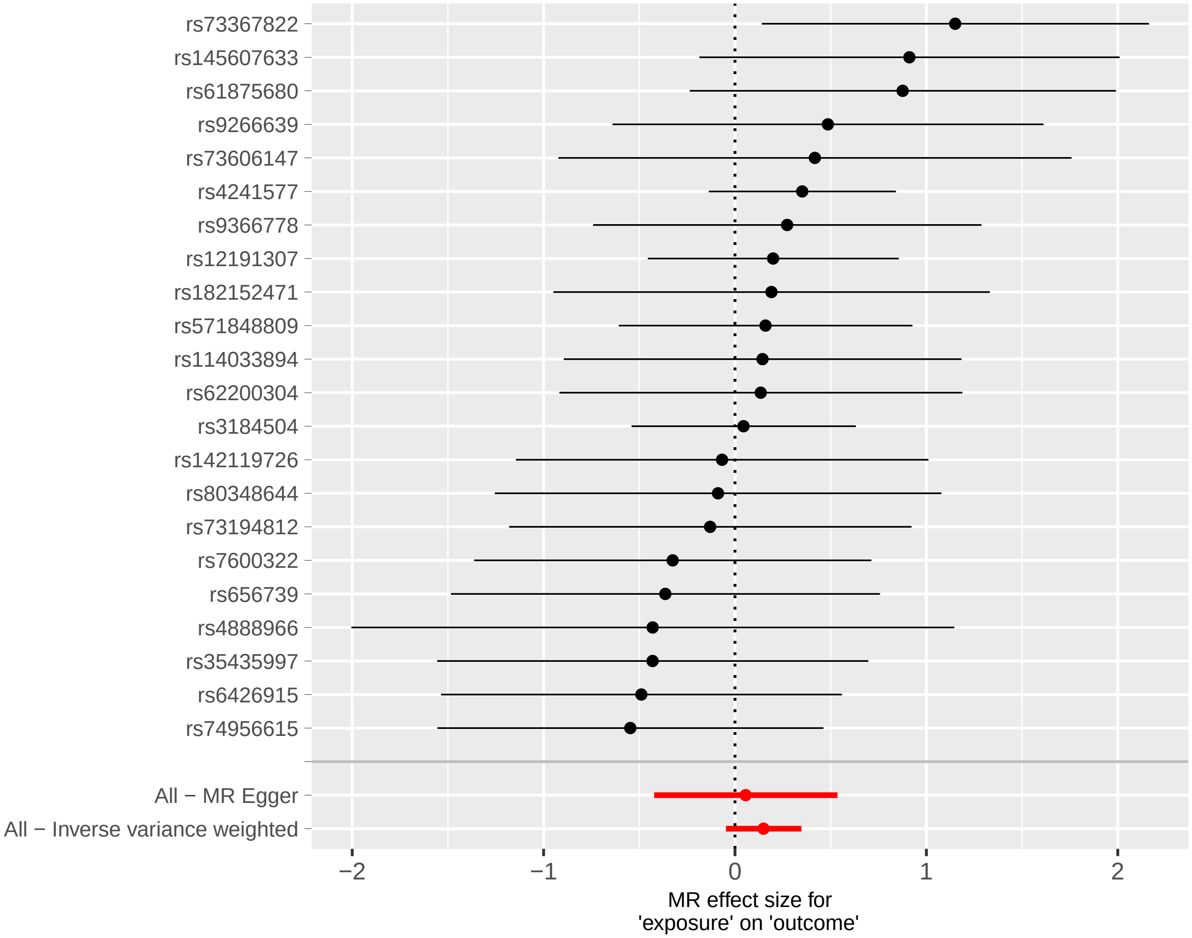
rs73367822
rs145607633
rs61875680
rs9266639
rs73606147
rs4241577
rs9366778
rs12191307
rs182152471
rs571848809
rs114033894
rs62200304
rs3184504
rs142119726
rs80348644
rs73194812
rs7600322
rs656739
rs4888966
rs35435997
rs6426915
rs74956615
All − MR Egger
All − Inverse variance weighted
−2
−1
0
1
2
MR effect size for
'exposure' on 'outcome'
